# P-935. Treatment and outcome characteristics in patients with *Staphylococcus aureus* Infective Endocarditis: A prospective observational study

**DOI:** 10.1093/ofid/ofae631.1126

**Published:** 2025-01-29

**Authors:** Gopal Krishana Bohra, Deepak Kumar, Navneet Kaur, Surender Deora, Rahul Choudhary, Naresh Kumar Midha, Durga Shankar Meena, Yash Khatod, Neetha T R, Tejasvi kanagiri, Vibhor Tak

**Affiliations:** All India Institute of Medical Sciences, Jodhpur, Jodhpur, Rajasthan, India; All India Institute of Medical Sciences, Jodhpur (India), Jodhpur, Rajasthan, India; All India Institute of Medical Sciences, Jodhpur, Jodhpur, Rajasthan, India; All India Institute of Medical Sciences, Jodhpur, Jodhpur, Rajasthan, India; All India Institute of Medical Sciences, Jodhpur, Jodhpur, Rajasthan, India; AIIMS, Jodhpur, Rajasthan, India; AIIMS, Jodhpur, Rajasthan, India; AIIMS Jodhpur, Jodhpur, Rajasthan, India; AIIMS Jodhpur, Jodhpur, Rajasthan, India; AIIMS, Jodhpur, Jodhpur, Rajasthan, India; AIIMS Jodhpur, Jodhpur, Rajasthan, India

## Abstract

**Background:**

Infective endocarditis (IE) is one of the most serious complication of *Staphylococcus aureus* bacteremia (SAB) and its incidence is on rise ranging from 10% to 46%.^1^ When present, it is linked to poor prognosis, with in-hospital mortality ranging from 20% to 30% highlighting to some extent the challenge in clinically diagnosing and treating the disease. ^2^There is lack of data focusing on the management of such cases in developing countries like India.

The present observational study was carried out to determine the treatment and outcome characteristics in patients with IE due to SAB.

**Figure:**
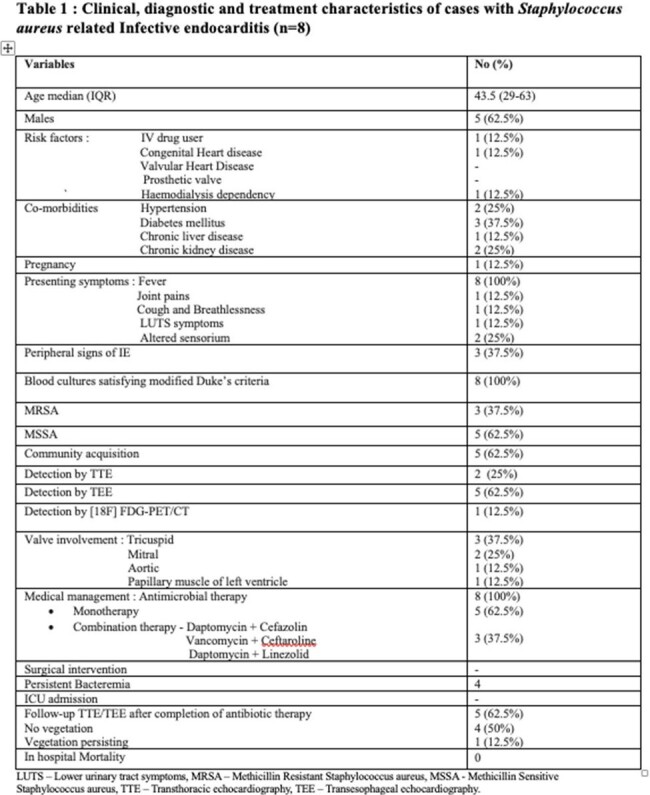

**Methods:**

This was a prospective observational study carried in Division of Infectious diseases, Department of General Medicine, All India Institute of Medical Sciences, Jodhpur between January 2023 to October 2023. All consecutive cases diagnosed in-hospital with SAB related IE using modified Duke’s criteria were enrolled in the study. The demographic and clinical details of each patient, the diagnostic evaluation, treatment given and outcomes were recorded. All these patients were followed at 1 month, 3 and 6 months after discharge.

**Figure:**
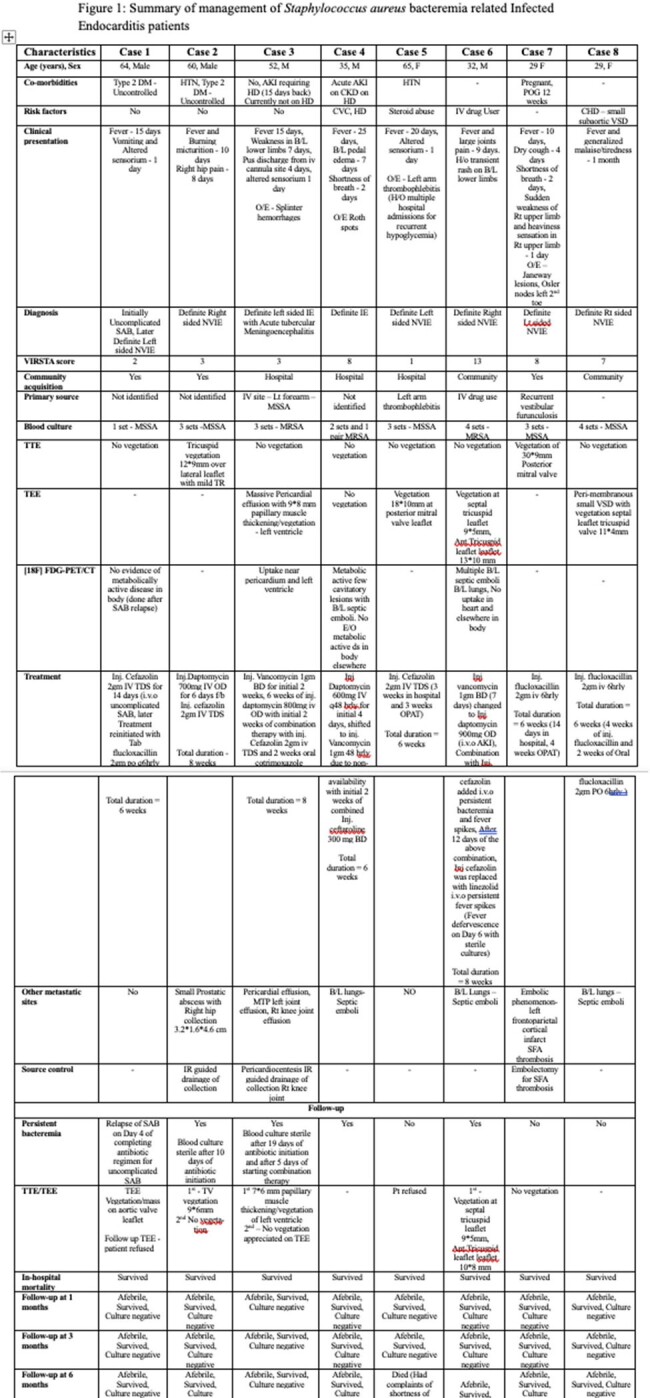

**Results:**

A total of eight cases of SAB related definite IE were included. The most common presenting symptom was fever, present in all cases. Peripheral signs of IE were observed in only three cases having splinter haemorrhages, Osler’s nodes, and Roth spots. IV drug use, congenital heart disease and hemodialysis dependency were found to be risk factors. All the eight patients received only medical management with 5 of them receiving antibiotic monotherapy and 3 of them receiving combination therapy. The reason for combination therapy was persistent bacteremia, lack of fever defervescence. The duration of therapy varied between 6-8 weeks. Five cases underwent repeat follow – up TEE/TTE revealing no vegetation in 4 cases and reduction of vegetation size in one case. There was no in-hospital mortality seen. At 6 months of follow-up one patient expired which was not attributable to SAB IE. Table 1 summarises the clinical, diagnostic and treatment characteristics of cases.

Figure 1 depicts detailed management of SAB related IE cases

**Conclusion:**

This study underscores multifaceted nature of this disease and the use of systematic approach for its management can improve the patient outcomes.

**Disclosures:**

**All Authors**: No reported disclosures

